# Clinical Impact of Rapid Reduction of Low-Density Lipoprotein Cholesterol Level on Long-Term Outcome of Acute Myocardial Infarction in the Statin Era: Subanalysis of the ALPS-AMI Study

**DOI:** 10.1371/journal.pone.0127835

**Published:** 2015-06-17

**Authors:** Takashi Miura, Atsushi Izawa, Hirohiko Motoki, Yusuke Miyashita, Yuichiro Kashima, Souichiro Ebisawa, Takeshi Tomita, Jun Koyama, Uichi Ikeda

**Affiliations:** Department of Cardiovascular Medicine, Shinshu University School of Medicine, Matsumoto, Japan; Showa University School of Pharmacy, JAPAN

## Abstract

**Background:**

The optimal period to achieve target percent reduction of low-density lipoprotein cholesterol (LDL-C) level for secondary prevention of acute myocardial infarction (AMI) is not well established.

**Methods:**

The Assessment of Lipophilic vs. Hydrophilic Statin Therapy in AMI (ALPS-AMI) study enrolled 508 patients (mean age, 66.0± 11.6 years; 80.6% male) who were hospitalized for AMI and underwent percutaneous coronary intervention (PCI). Of these patients, 81 were excluded because of the absence of LDL-C measurements at 4 weeks after randomization. In the remaining 427 patients, the target LDL-C level reduction of ≥30% was achieved and not reached within 4 weeks after randomization in 204 cases (early reduction group) and 223 cases (late reduction group). The groups were formed prospectively and analyzed with regard to the composite end point (major adverse cardiovascular event [MACE]: all-cause death, myocardial infarction, and stroke) and clinical outcomes.

**Results:**

MACE were significantly more frequent in the late reduction group compared to the early reduction group (9.4% vs. 3.4%, P = 0.013). The incidence of cardiac deaths was also significantly higher in the late reduction group (3.1% vs. 0.5%, P = 0.044). On age-adjusted Cox proportional hazards analysis in statin-naïve patients, percent reduction of LDL-C level during the initial 4 weeks (HR, 0.98; 95% CI: 0.97–0.99, P = 0.042) and baseline LDL-C level (HR, 0.98; 95% CI: 0.97–0.99, P = 0.033) predicted adverse events.

**Conclusions:**

Rapid reduction of LDL-C level is strongly associated with favorable outcome in patients with AMI.

## Introduction

Dyslipidemia is a major adverse risk factor for the development of coronary artery disease, and cholesterol level control with statins has been shown to be beneficial in both primary and secondary prevention of coronary artery disease (CAD) [1, 2]. Recently, several studies revealed significant clinical benefits of intensive reduction of serum low-density lipoprotein cholesterol (LDL-C) level in patients with coronary artery disease for prevention of cardiovascular events. Prior ACC/AHA and ESC guidelines suggested the achievement of a target LDL-C level as the optimal goal of LDL-C management in secondary prevention of acute myocardial infarction (AMI) [3, 4]. However, the Cholesterol Treatment Trialists`(CTT) Collaboration, a meta-analysis of data from 169,138 individuals in 26 randomized trials, suggested that percent reduction of LDL-C is more important for secondary prevention than achievement of a target LDL-C level in patients with coronary artery disease [5]. Accordingly, the recent 2013 ACC/AHA Guideline recommend using high-intensity statin therapy to achieve a percent reduction of LDL-C level instead of a target LDL-C level for secondary prevention [6], with high-intensity and moderate-intensity statin therapies defined as daily doses that lower LDL-C by ≥50% or between 30% and 50%, respectively. All patients with atherosclerotic cardiovascular disease and no safety concerns should receive high-intensity statin therapy; whereas moderate-intensity therapy can be used if safety concerns are present. In 2012, Japan Atherosclerosis Society Guidelines for prevention of atherosclerotic cardiovascular disease recommended the target LDL-C level of less than 100 mg/dL for secondary prevention and stated that a 20 to 30% decrease in LDL-C level could reduce coronary artery events by approximately 30%. Because of the stricter regulatory requirements, the maximum allowed statin doses in Japan are comparable with the moderate-intensity doses from the 2013 ACC/AHA Guideline. Importantly, the optimal period to achieve a target percent reduction of LDL-C level in patients with AMI has not yet been well established. Based on the aforementioned differences in clinical guidelines and statin usage, we hypothesized that rapid LDL-C reduction by ≥ 30% after percutaneous coronary intervention (PCI) might be beneficial, in terms of clinical outcomes. Accordingly, this study evaluated long-term clinical outcomes of AMI patients who had been treated with moderate-intensity doses of statins within 96 hours after PCI.

## Methods

### Study design

The ALPS-AMI (Assessment of Lipophilic vs. Hydrophilic Statin Therapy in Acute Myocardial Infarction) study was a prospective, multicenter, randomized, open-labeled study designed to provide up to 24 months of clinical follow-up [7]. Briefly, men and women at least 20 years old hospitalized with ST segment elevation MI (STEMI) or non ST-segment elevation MI, were randomly allocated within 96 hours of their PCI to receive either 10mg of atorvastatin or 10mg of pravastatin daily. The protocol required the baseline serum LDL-C level to be >70 mg/dL. If the LDL-C level was > 100 mg/dL after a 4-week statin treatment, the dose was increased to 20 mg daily. All patients were pretreated with clopidogrel (75 mg daily) in addition to aspirin (100–200 mg daily). A loading dose of 300 mg of clopidogrel was administered to clopidogrel-naïve patients. The interventional strategy and stent selection were left to the discretion of the operator in all procedures. Exclusion criteria included planned surgery for coronary artery bypass grafting, pregnancy, active liver or renal diseases, malignant diseases, withdrawal of informed consent, and serious arrhythmic events or the presence of hemodynamic instabilities (hypotension, congestive heart failure, or mechanical complications following AMI).

**Fig 1:**
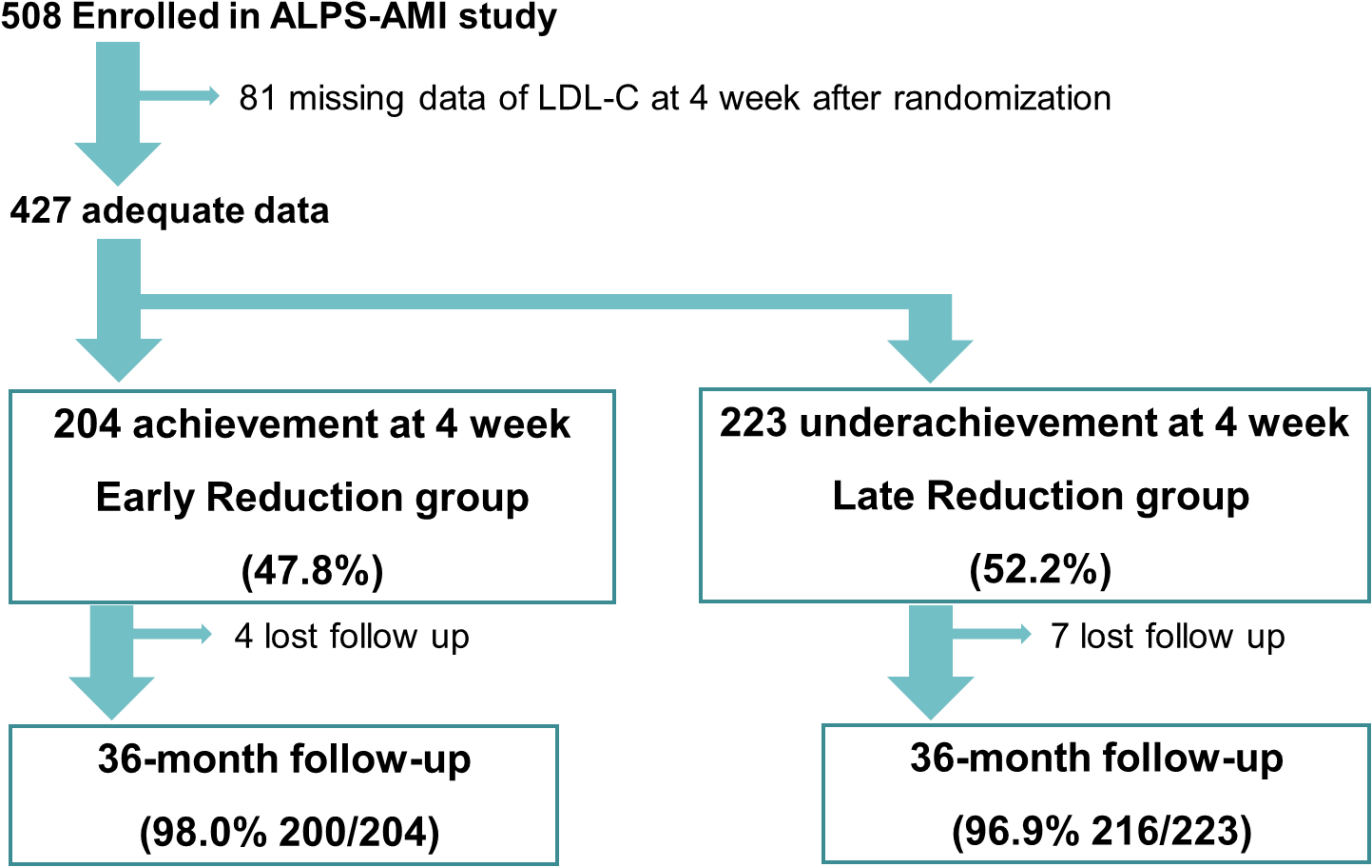
Study flowchart.

Between June 2008 and December 2010, a total of 508 patients enrolled in the ALPS-AMI study. Of these patients, 81 were excluded because of the absence of LDL-C measurements at 4 weeks after randomization. In the remaining 427 patients, the LDL-C level reduction of ≥ 30% was achieved and not reached within 4weeks after randomization in 204 (early reduction group) and 223 (late reduction group) cases, respectively. The 2 groups were formed prospectively and analyzed with regard to primary and secondary outcomes (Fig 1). The follow-up period was 36 months.

Patients were enrolled upon providing written informed consent. The protocol of this study was approved by the hospital ethics committee, and the study was performed in accordance with the Declaration of Helsinki. Ethics committee approval was obtained from the Committee for Medical Ethics of Shinshu University School of Medicine (1019). The ALPS-AMI study was registered with the University Hospital Medical Information Network-Clinical Trials Registry (UMIN-CTR), as accepted by the International Committee of Medical Journal Editors (No. UMIN 000001521).

### Definitions and outcomes

AMI was diagnosed according to the AHA/ACC guidelines [8]. The primary outcome measure was a composite of major adverse cardiovascular events (MACE; all-cause death, myocardial infarction [MI], and stroke). The secondary outcome measure endpoints were cardiovascular death and hospitalization because of congestive heart failure.

Angiographic success was defined as achievement of a minimum stenosis diameter reduction to less than 20% with grade 3 Thrombolysis In Myocardial Infarction (TIMI) flow. Coronary artery disease was defined as >50% stenosis in a coronary vessel on angiography, history of coronary artery bypass graft surgery or PCI, or previous MI. Stroke was defined as ischemic stroke that persisted for ≥24 h and was diagnosed by a neurologist. Body mass index was defined as weight in kilograms divided by the square of height in meters. Hypertension was defined as systolic blood pressure (BP) ≥140 mmHg, diastolic BP ≥90 mmHg, or ongoing therapy for hypertension. Hypercholesterolemia was defined as a serum total cholesterol concentration ≥220 mg/dl, a low-density lipoprotein-cholesterol concentration ≥140 mg/dl, or current treatment with lipid-lowering therapy. Diabetes was defined as HbA1c ≥6.5%, casual plasma glucose ≥200 mg/dl or treatment with oral hypoglycemic agents or insulin injection. Left ventricular ejection fraction (LVEF) was measured by echocardiography and LVEF ≤40% indicated LV dysfunction. Multi-vessel disease was defined by the presence of a ≥75% lesion in ≥2 major coronary arteries. Family history of CAD defined as CAD in a first-degree relatives.

### Statistical analysis

Continuous variables are reported as mean ± standard deviation. Categorical variables are reported as frequencies and percentages. Characteristics of the patients in the 2 groups were compared using the chi-squared test. Continuous variables were compared using the t-test. For each group, multivariate Cox proportional hazard models were used to adjust for the effects of baseline risk factors on major adverse cardiovascular events. P < 0.05 was considered to indicate a statistically significant difference. Statistical analyses were performed with IBM SPSS Statistics Version 21 software.

## Results

### Baseline demographics

The early reduction group had a significantly younger and higher rate of hypertension, smoking, and lower rate of administration of statins at admission compared to the late reduction group. The 2 groups were similar with respect to the gender distribution (79.9% vs. 80.7% males, P = 0.83, respectively). With regard to medication, the patients in the early reduction group were administered atorvastatin predominantly than those in the late reduction group (70.6% vs. 29.6%, P < 0.0001). Regarding PCI procedure and lesions, all coronary artery stenting was performed by bare metal stent (BMS) in almost cases with approximately the same frequency in both groups. There was no left main trunk (LMT) lesions, because of worse prognosis of them. The characteristics of the patients and lesion at admission and medication at discharge are shown in Table 1.

**Table 1:** Patient Characteristics.

Patient Characteristics	Overall	Early Reduction	Late Reduction	P value
N	427	204	223	
Age (years)	65.8 ± 11.5	64.6 ± 10.8	67.0 ± 12.0	0.029
Male	343 (80.3)	163 (79.9)	180 (80.7)	0.83
BMI (Kg/m^2^)	23.7 ± 3.8	24.0 ± 3.9	23.4 ± 3.7	0.093
Hypertension	184 (43.1)	98 (48.0)	85 (35.9)	0.042
Hypercholesterolemia	159 (37.2)	96(47.1)	62 (27.8)	<0.0001
Baseline LDL (mg/dL)	131.4 ± 33.9	141.5 ± 34.6	121.4 ± 28.8	<0.0001
Diabetes	105 (24.6)	55 (27.0)	50 (22.4)	0.28
Smoking	274 (64.2)	146 (71.6)	128 (57.4)	0.002
History of CAD	32 (7.5)	14 (6.9)	19 (8.5)	0.52
History of stroke	25 (5.9)	12 (5.9)	13 (5.8)	0.98
LV dysfunction	42 (9.8)	24 (11.8)	18 (8.1)	0.32
Multivessel disease	138 (32.3)	71 (34.8)	66 (29.6)	0.25
Family history of CAD	94 (22.0)	42 (20.6)	51 (22.9)	0.57
eGFR (mL/min/1.73 m^2^)	71.5 ± 19.4	72.4 ± 19.4	70.6 ± 19.4	0.33
Previous administration of statins	62 (14.5)	19 (9.3)	43 (19.3)	0.003
Index presentation				
STEMI at admission	352 (82.4)	165 (80.9)	187 (83.9)	0.42
Killip class Ⅰ	382 (89.5)	184 (90.2)	198 (88.8)	0.76
Culprit lesion				
LAD	200 (46.8)	103 (50.5)	98 (44.0)	0.18
LCx	67 (15.7)	28 (13.7)	39 (17.4)	0.29
RCA	165 (38.6)	77 (37.8)	87 (39.0)	0.79
PCI procedure				
BMS use	411 (96.2)	198 (97.1)	213 (95.5)	0.48
POBA alone	16 (3.8)	6 (2.9)	10 (4.5)	0.48
Medication at discharge				
ACEIs/ARBs	368 (86.2)	176 (86.2)	192 (86.1)	0.96
CCBs	76 (17.8)	44 (21.6)	32 (14.4)	0.051
ß-blockers	249 (58.3)	120 (58.8)	130 (58.3)	0.96
Warfarin	3 (0.7)	1 (0.5)	2 (0.1)	0.62
Atorvastatin	210 (49.2)	144 (70.6)	66 (29.6)	<0.0001

Data are presented as n (%) or mean ± SD. LV dysfunction was defined as <40% of LV ejection fraction. BMI, body mass index; CAD, coronary artery disease; STEMI, ST elevation myocardial infarction; eGFR, estimated glomerular filtration rate, LAD, left ascending artery; LCx, left circumflex artery; RCA, right coronary artery; BMS, bare metal stent; POBA, plain old balloon angioplasty; ACEIs, angiotensin-converting enzyme inhibitors; ARBs, angiotensin receptor blockers; CCBs, calcium-channel blockers; LV, left ventricular.

### Changes of the LDL-C level

**Fig 2:**
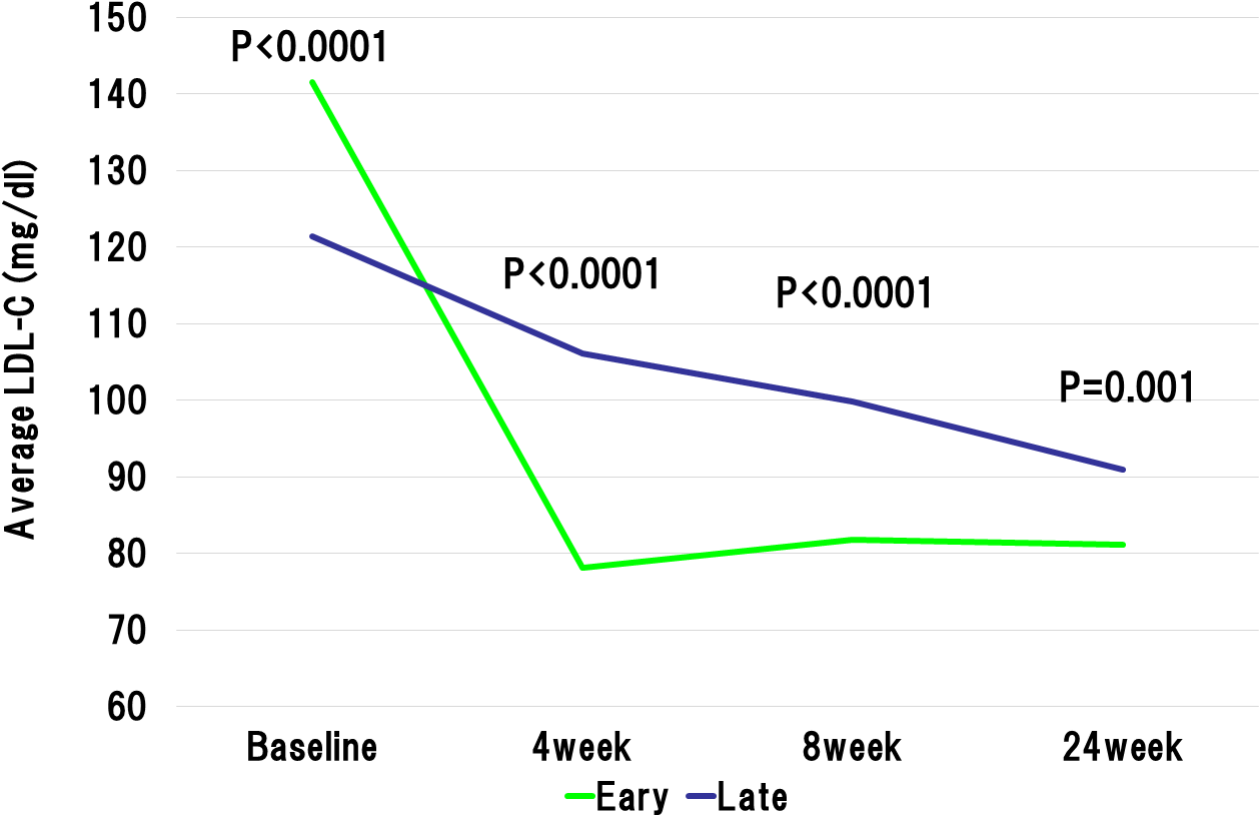
Changes in low-density lipoprotein (LDL) cholesterol level in early or late reduction groups. Although baseline LDL was markedly higher in the early reduction group, at the 4, 8, and 24 weeks after the beginning of statin administration, LDL-C levels was significantly lower in the early reduction group than in the late reduction group.

**Fig 3:**
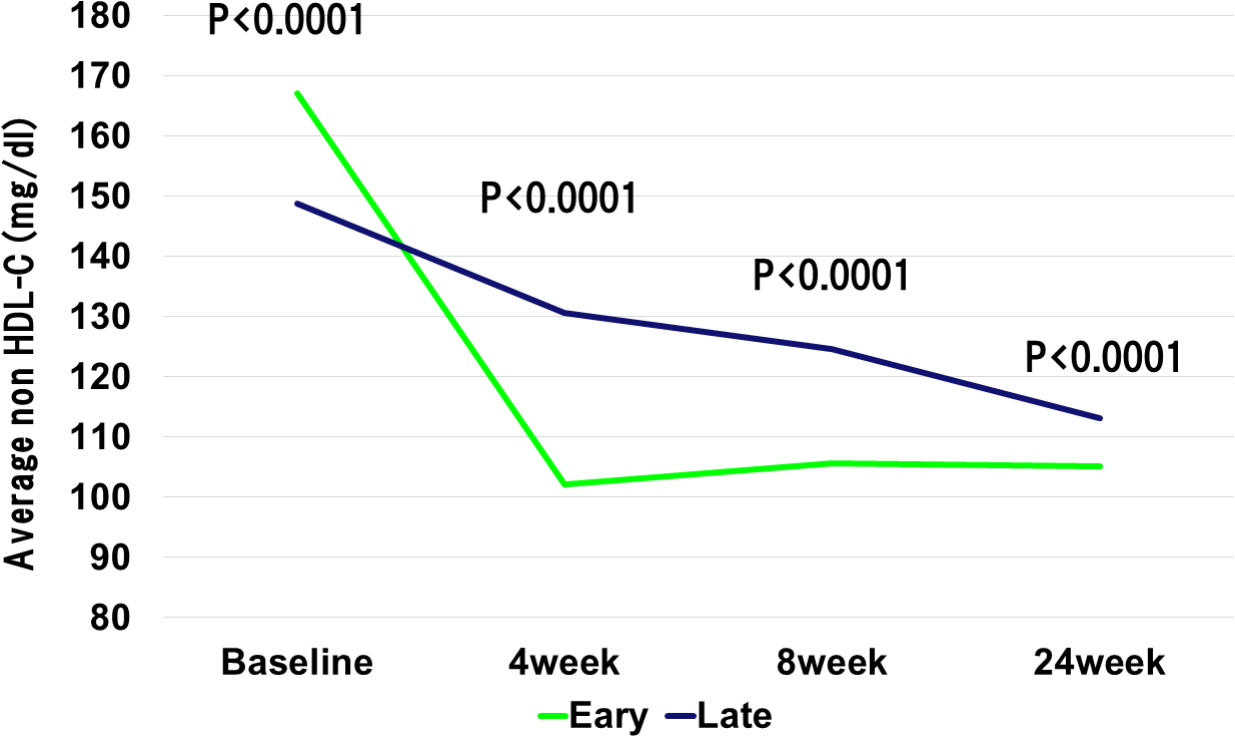
Changes in non high-density lipoprotein (non HDL) cholesterol level in early or late reduction groups. Change of non HDL in two groups were similar with change of LDL-C levels.

**Fig 4:**
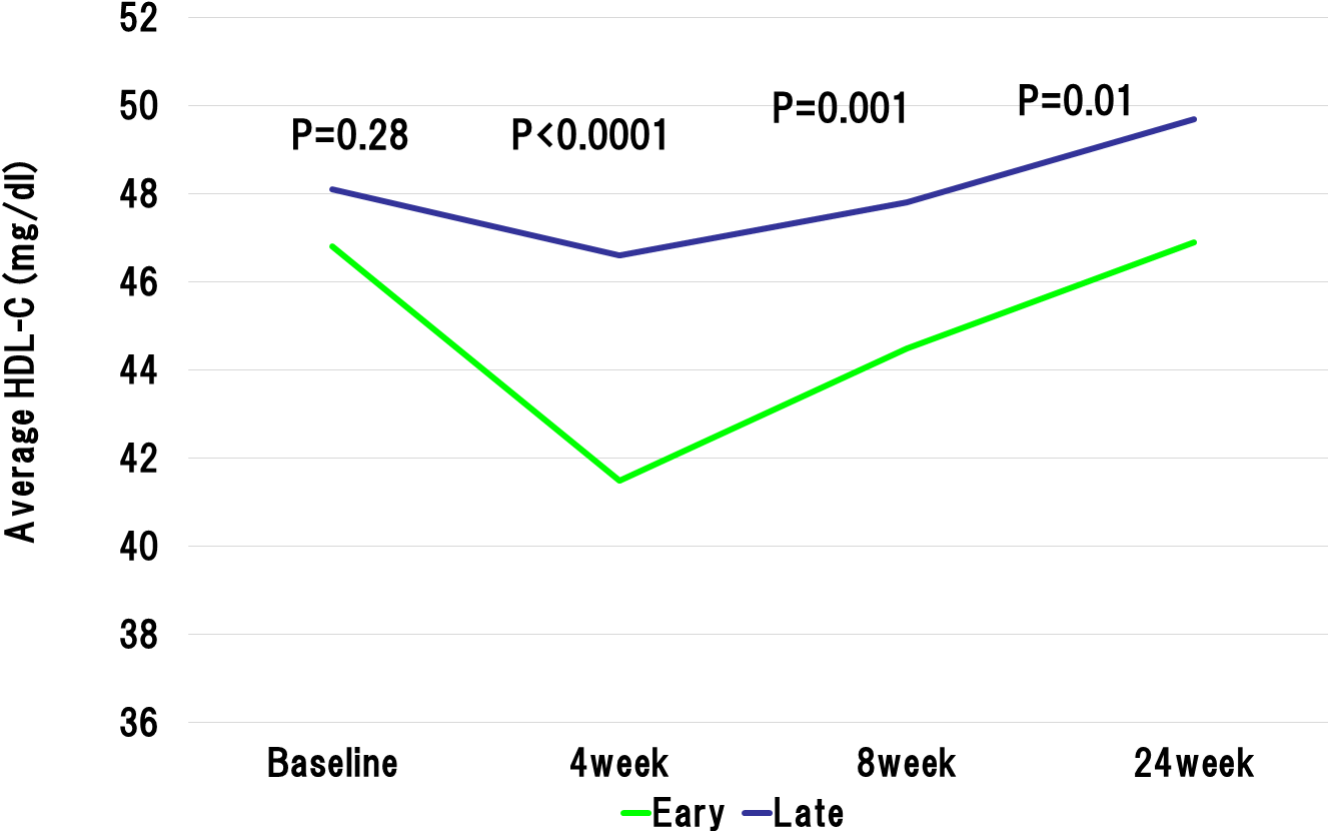
Changes in high-density lipoprotein (HDL) cholesterol level in early or late reduction groups. Baseline HDL-C level was similar in the both groups. However, the HDL-C levels at 4 weeks, 8 weeks, and 24 weeks after randomization were significantly lower in the early reduction group than late reduction group.

**Fig 5:**
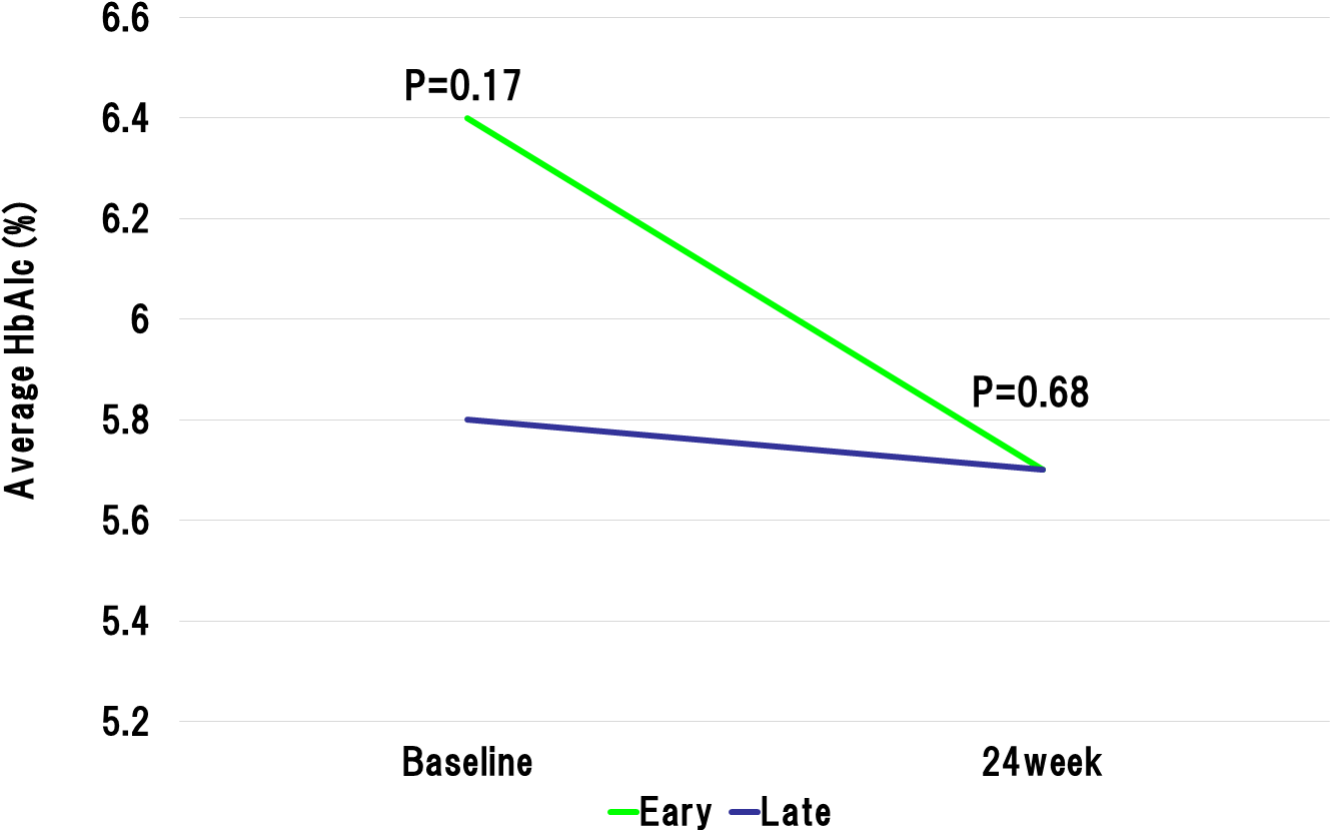
Changes in hemoglobin A1c (HbA1c) level in early or late reduction groups. At baseline and 24 weeks after randomization, there were no significant difference between early and late reduction groups.

The baseline LDL-C levels in the early reduction group was significantly higher than those in the late reduction group. However, at 4weeks after the initiation of statin administration, the LDL-C levels were significantly lower in the early reduction group than late reduction group (78.1 ± 20.3 mg/dL vs. 106.1 ± 26.9 mg/dL, P < 0.0001). The statin dose was increased in accordance with the ALPS AMI protocol in the patients with LDL-C level ≥ 100mg/dL at 4 weeks after randomization. At the 8 weeks after the beginning of statin administration, LDL-C levels still showed significant difference between the early and late reduction groups (81.8 ± 20.9 mg/dL vs. 99.8 ± 27.3 mg/dL, P < 0.0001) (Fig 2). Importantly, the early reduction group revealed the absolute reductions in the LDL-C levels during initial 4 weeks and 8 weeks were significantly higher than the late reduction group (63.4 ± 23.9 mg/dL vs. 15.3 ± 19.3 mg/dL, P < 0.0001, and 59.7 ± 28.4 mg/dL vs. 22.0 ± 24.0 mg/dL, P < 0.0001, respectively). Furthermore, relative reductions in the LDL-C levels during initial 4 weeks and 8 weeks were significantly higher in the early reduction groups than those in the late reduction group (44.2 ± 9.5% vs. 11.7 ± 15.8%, P < 0.0001, and 41.0 ± 12.9% vs. 17.0 ± 18.6%, P < 0.0001, respectively). Similarly, absolute reductions in the non HDL-C levels during initial 4 weeks and 8 weeks were significantly higher than the late reduction group (Fig 3). With regard to high-density lipoprotein cholesterol (HDL-C), baseline HDL-C level was similar in the both groups. However, the HDL-C levels at 4 weeks and 8 weeks after randomization were significantly lower in the early reduction group than late reduction group (41.5 ± 9.7 mg/dL vs. 46.6 ± 10.5 mg/dL, P < 0.0001, and 44.5 ± 10.1 mg/dL vs. 47.8 ± 12.2 mg/dL, P = 0.001, respectively) (Fig 4). Regarding HbA1c at baseline and 24 weeks after randomization, there were no significant difference between early and late reduction groups (Fig 5).

At the end of study, there was significant difference in patients with administration 20mg of atorvastatin or pravastatin between early and late reduction groups (30 [14.7%] vs. 121 [54.3%], P < 0.0001). Regarding other lipid lowering drugs, use of ezetimibe was significantly lower in early reduction group than those in late reduction group (14 [6.9%] vs. 40 [17.9%], P < 0.0001).

### Endpoints

**Fig 6:**
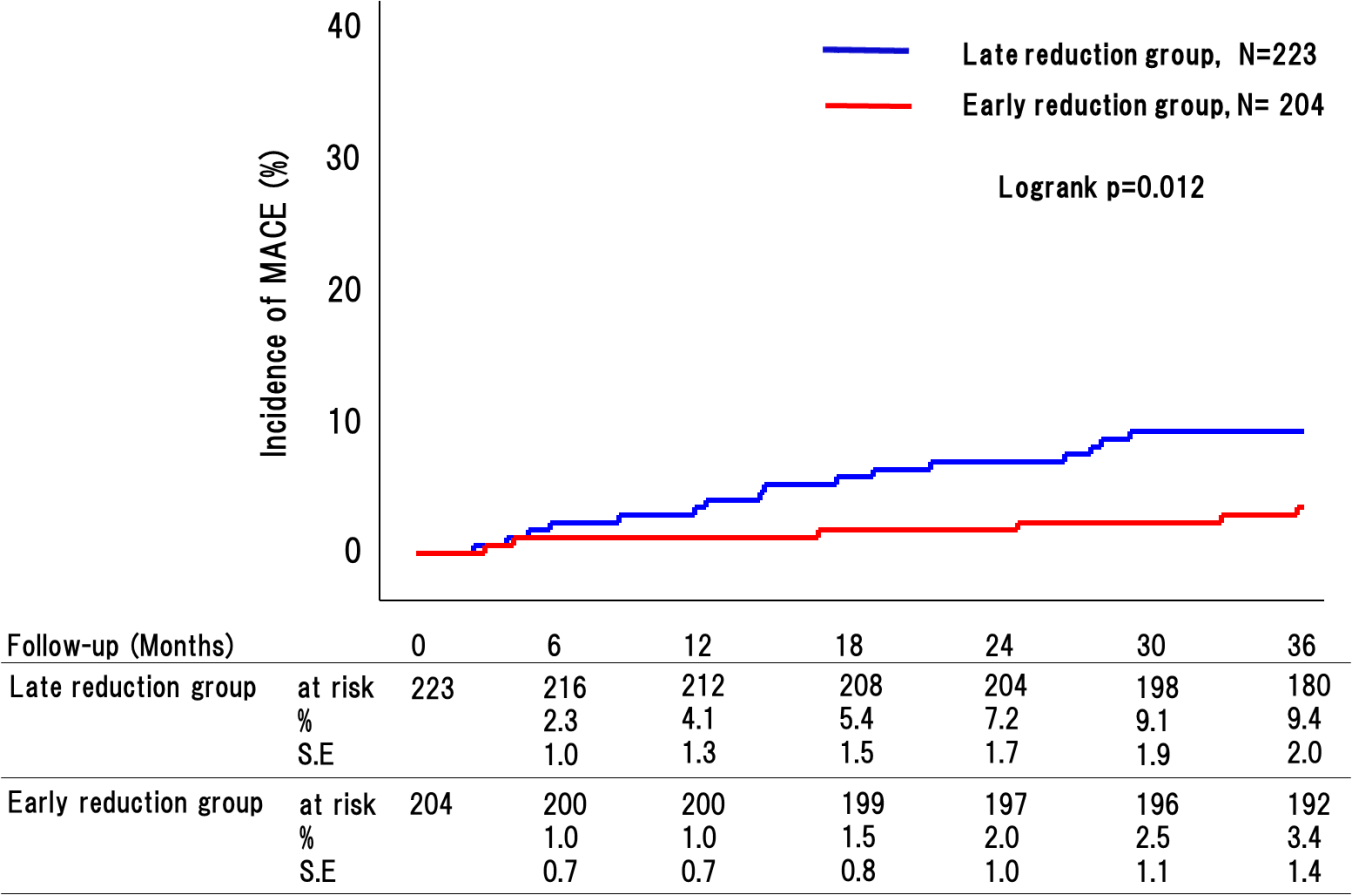
Incidence of major adverse cardiovascular events (MACE; includes all-cause death, myocardial infarction, and stroke) between patients in late reduction group and early reduction group. On intention-to-treat analysis, the incidence of MACE was significantly higher in the late reduction group (9.4% vs. 3.4%, P = 0.012).

Clinical follow-up was completed in 416 out of 427 patients (97.4%), with the 11 remaining patients dropping out during the follow-up period. MACE occurred in 28 patients (7 [3.4%] in the early reduction group and 21 [9.4%] in the late reduction group [P = 0.013].) (Fig 6). There were 22 instances of deaths (6 [2.9%] in the early reduction group and 16 [7.2%] in the late reduction group [P = 0.048]), including cardiac death in 8 cases (1 [0.5%] in the early reduction group and 7 [3.1%] in the late reduction group [P = 0.044] (Table 2). Non-cardiac death included intracranial bleeding in 3 case (1 [0.5%] in the early reduction group and 2 [0.9%] in the late reduction group [P = 0.62]), pneumonia in 1 case (1 [0.5%] in the early reduction group), malignancy in 2 case (2 [4.6%] in the late reduction group), unknown in 5 cases (3 [1.5%] in the early reduction group and 3 [1.3%] in the late reduction group [P = 0.91]). MI occurred in 2 patients (2 [0.9%] in the late reduction group), and stroke occurred in 6 patients (1 [0.5%] in the early reduction group and 5 [2.2%] in the late reduction group [P = 0.12]), and Heart failure occurred in 10 cases (5 [2.5%] in the early reduction group and 5 [2.2%] in the late reduction group [P = 0.89]) (Table 2).

**Table 2:** Event rate between early and late reduction group.

	Overall	Early Reduction	Late Reduction	P value
N	427	204	223	
MACE	28 (6.6)	7 (3.4)	21 (9.4)	0.013
All-cause death	22 (5.2)	6 (2.9)	16 (7.2)	0.048
Cardiovascular death	15 (3.5)	2 (1.0)	10 (4.5)	0.029
Cardiac death	11 (2.6)	1 (0.5)	7 (3.1)	0.044
MI	2 (0.5)	0 (0.0)	2 (0.9)	0.18
Stroke	6 (1.4)	1 (0.5)	5 (2.2)	0.12
TLR	98 (23.0)	43 (21.1)	55 (24.7)	0.38
Hospitalization due to CHF	10 (2.3)	5 (2.5)	5 (2.2)	0.89

MACE was defined as all-cause death, MI, or stroke. MACE, major adverse cardiovascular events; Cardiac death was included any deaths with an immediate cardiac cause, sudden deaths with unknown cause. Cardiovascular death was included any deaths with an immediate vascular cause, and cardiac death. MI, myocardial infarction; TLR, target lesion revascularization; CHF, congestive heart failure.

Regarding in-hospital sustained ventricular tachycardia or ventricular fibrillation, there were similar frequency in early and late reduction group (8 [3.9%] vs. 10 [4.5%], P = 0.79).

Age-adjusted Cox proportional hazards analysis was performed to evaluate the effects of the rapid reduction of LDL-C and other variables on MACE (Table 3). The percent reduction of the LDL-C levels during the initial 4 weeks didn`t predict adverse events after adjustment for age (Table 4).

**Table 3:** Univariate predictors of MACE.

	Unadjusted HR (95% CI)	P value
Variables		
Female	0.68 (0.323–1.96)	0.48
Age	1.06 (1.00–1.10)	0.003
⊿LDL-C during 4 weeks	0.98 (0.97–0.99)	0.007
% reduction of LDL during 4 weeks	0.98 (0.97–0.99)	0.0021
Baseline LDL-C	0.98 (0.97–0.99)	0.024
Baseline HDL-C ≤40 md/dL	0.77 (0.31–1.91)	0.57
LDL-C at 4 weeks	1.00 (0.98–1.01)	0.98
LDL-C at 8 weeks	0.99 (0.97–1.01)	0.16
⊿LDL-C during 8 weeks	0.99 (0.98–1.01)	0.29
% reduction of LDL during 8 weeks	1.00 (0.98–1.02)	0.99
LDL-C < 100 mg/dL at 4 weeks	0.96 (0.44–2.07)	0.91
LDL-C < 70 mg/dL at 4 weeks	1.12 (0.45–2.64)	0.79
Hypertension	0.74 (0.34–1.60)	0.44
Diabetes	1.04 (0.44–2.43)	0.94
HbA1c ≤8.0%	0.63 (0.85–4.62)	0.65
STEMI	0.63 (0.27–1.48)	0.29
eGFR	0.97 (0.95–0.99)	0.001
Atorvastatin	0.55 (0.25–1.19)	0.13

CI, confidence interval; HR, hazard ratio; MACE, major adverse cardiovascular events; STEMI, ST elevation myocardial infarction; eGFR, estimated glomerular filtration rate.

**Table 4:** Age-adjusted Cox proportional hazard analysis of MACE.

	Adjusted HR (95% CI)	P value
Variables		
Female	0.54 (0.15–1.95)	0.34
⊿LDL-C during 4 weeks	0.99 (0.97–1.00)	0.08
% reduction of LDL during 4 weeks	0.98 (0.96–1.00)	0.095
Baseline LDL-C	0.99 (0.98–1.01)	0.27
Baseline HDL-C ≤40 md/dL	0.72 (0.29–1.78)	0.47
LDL-C at 4 weeks	1.04 (0.99–1.02)	0.51
LDL-C at 8 weeks	1.01 (0.99–1.02)	0.49
⊿LDL-C during 8 weeks	0.99 (0.98–1.01)	0.30
LDL-C ≤100 mg/dL at 4 weeks	0.73 (0.28–1.91)	0.53
LDL-C ≤70 mg/dL at 4 weeks	0.81 (0.29–2.65)	0.81
Hypertension	0.58 (0.20–1.64)	0.30
Diabetes	1.09 (0.46–2.57)	0.84
HbA1c ≥8.0%	0.67 (0.91–4.92)	0.69
STEMI	0.41 (0.15–1.08)	0.071
eGFR	0.96 (0.94–0.99)	0.002
Atorvastatin	0.38 (0.14–1.07)	0.066

CI, confidence interval; HR, hazard ratio; MACE, major adverse cardiovascular events; STEMI, ST elevation myocardial infarction; eGFR, estimated glomerular filtration rate.

### Analysis in statin naïve patients

In present study, statin naïve patients were 365 out of 427 (85.5%). We performed age-adjusted Cox proportional hazards analysis to evaluate the pure effects of the rapid reduction of LDL-C on MACE in statin naïve patients. The percent reduction of the LDL-C levels during the initial 4 weeks and the baseline LDL-C levels were significant predictor after adjustment for age (HR, 0.98; 95% CI: 0.97–0.99, P = 0.042, HR, 0.98; 95% CI: 0.97–0.99, P = 0.033, respectively). However, the percent reduction of the LDL-C levels during the initial 8 weeks and the percent reduction of the LDL-C levels ≥ 30% during the initial 8 weeks were not significant predictor after adjustment for age (HR, 0.99; 95% CI: 0.98–1.02, P = 0.83, HR, 1.12; 95% CI: 0.50–2.50, P = 0.78, respectively). Furthermore, the LDL-C ≤100mg/dL and the LDL-C ≤70mg/dL at 4 weeks after randomization were not also associated with adverse events after adjustment for age (HR, 0.80; 95% CI: 0.40–1.88, P = 0.61, HR, 0.91; 95% CI: 0.36–2.29, P = 0.84, respectively).

### Comparisons between achievement and non-achievement of LDL-C late reduction

In our study, the LDL-C level reduction of ≥ 30% was achieved and not reached within 8weeks after randomization in 248 (achievement of late reduction group) and 179 (non-achievement of late reduction group) cases, respectively. Incidence of MACE was similar between achievement of late reduction group and non-achievement of late reduction group (6.9% vs. 6.3%, P = 0.811). Furthermore, all-cause mortality, incidence of MI and stroke were similar between two groups (5.2% vs. 5.1%, P = 0.968, 0.4% vs. 0.6%, P = 0.805, 1.2% vs. 1.7%, P = 0.666, respectively).

### Efficacy of percent reduction of LDL-C between atorvastatin and pravastatin groups

MACE rate was similar between patients with atorvastatin and those with pravastatin (95.2% vs. 91.7%, P = 0.12). In atorvastatin groups, MACE rate was no significant difference between early and late reduction groups (96.5% vs. 92.4%, P = 0.21). Furthermore, in pravastatin groups, MACE rate was also similar between early and late reduction groups (96.7% vs. 89.8%, P = 0.096). However, sample size of these analysis were relatively small to evaluate efficacy of rapid reduction of LDL-C level in each statin group.

## Discussion

Many randomized trials compared more and less intensive statin therapies as well as statin and control patients groups to elucidate the effectiveness of primary and secondary prevention of atherosclerotic cardiovascular diseases. Previous studies have already revealed that intensive statin lipid-lowering therapy reduce the recurrence of ischemic events in patients with AMI [9, 10]. However, it is still unclear whether achieving a large or rapid reduction of the LDL-C level is more important. Therefore, we evaluated the long-term clinical outcomes of AMI patients after PCI depending on the time period needed to achieve the target LDL-C percent reduction (≥ 30%) with moderate-intensity statin therapy.

### Changes of the LDL-C level

Previous reports have suggested that higher baseline LDL-C levels are associated with favorable clinical outcomes after PCI in patients with AMI (the cholesterol paradox) [11].

In the current study, the baseline LDL-C levels and percent reduction of the LDL-C levels during the initial 4 weeks were not associated with adverse events in overall analysis including the patients with prior statin therapy. However, these were independent predictors for adverse events in stain naïve patients alone. The design of many previous study excluded the patients with prior statin therapy or required those to have higher cholesterol level. In current study, there were no exclusion criteria for patients with prior statin therapy who had already lowered LDL-C level. Therefore, although overall analysis didn`t show the efficacy of rapid reduction of LDL-C level on adverse events, statin-naïve patients analysis revealed the efficacy of those on adverse events.

Furthermore, although the patients with higher baseline LDL-C levels had larger initial reduction of LDL-C level in overall analysis, statin-naïve patients analysis showed these phenomenon was more pronounced. Therefore, higher LDL-C at baseline was associated with lower event rates in statin-naïve patients analysis.

### Target percent reductions in LDL-C and the periods needed to achieve them

In statin-naïve patients analysis, age-adjusted Cox proportional hazards analysis revealed that percent reduction of LDL-C levels during the initial 4 weeks were independent predictors of adverse events, but percent reduction and range of LDL-C reduction during initial 8 weeks and the achievement of LDL-C percent reduction ≥ 30% during 8weeks were not. This result demonstrates that the achieving a target percent reduction in LDL-C during the initial 4 weeks is significantly associated with favorable prognosis in patients with AMI. Furthermore, in age-adjusted Cox proportional hazards analysis, although the LDL-C ≤100mg/dL and the LDL-C ≤70mg/dL at 4 weeks after randomization were not independent predictors of adverse events, the percent reduction of LDL-C levels during the initial 4 weeks was independent predictor of them. This result might suggest that aggressive LDL-C reduction by percent reduction of LDL-C was more important than by target LDL-C levels for secondary prevention in patients with AMI.

Early statin treatment has been shown to decrease long-term mortality and subsequent cardiac events in patients with AMI [12]. In addition, the PROVE IT-TIMI22 trial revealed that intensive statin therapy reduced C-reactive protein levels more significantly than moderately intensive statin therapy in patients with AMI [13]. Another study showed that intensive statin therapy reduced coronary plaque, and this reduction was associated with anti-inflammatory effects of statins [14]. Moreover, a prior report found that rapid reductions in fluorodeoxyglucose uptake detected by fluorodeoxyglucose-positron emission tomography/ computed tomographic imaging may represent changes in atherosclerotic plaque inflammation that occur as early as 4 weeks after the initiation of statin therapy [15]. These previous studies support the critical role of achieving a target percent reduction of the LDL-C level within the initial 4 weeks after AMI revealed in the present study. Whereas, Vale N et al previously reported that initiation of statin therapy within 14 days following ACS does not reduce death, myocardial infarction, or stroke up to four months, but reduces the occurrence of unstable angina at four months following ACS [16]. In current study, early reduction group had significantly lower mortality and rate of cardiac death than late reduction group. This discrepancy may teach us that the early achieving a target percent reduction in LDL-C was more important than only early initiation of statin therapy. Furthermore, in Comparisons between achievement and non-achievement of LDL-C percent reduction ≥ 30%, we showed similar adverse events rate in two groups. This results suggested early achieving a target percent reduction in LDL-C was more important than late achieving therapy.

Regarding MACE rate, those in the late reduction group was comparable to previous reports. A 6.4% major adverse events which including MI, stroke, cardiovascular death at 2 years were reported from Pacific registry [17]. The Heart Institute of Japan Acute Myocardial Infarction (HIJAMI) registry showed 9.4% in-hospital mortality and 15.9% post-discharge mortality at 4.3 years [18]. In current study, although late reduction group had comparable outcomes, the percent reduction of the LDL-C levels ≥ 30% during the initial 8 weeks was not an independent predictor.

### Other predictor of adverse events

Many previous report revealed renal insufficiency was strong predictor of adverse ischemic events in patients with AMI [19, 20]. In current study, estimated glomerular filtration rate (eGFR) was an independent predictor of MACE after adjustment for age. Previous studies showed the presence of diabetes was the important risk factor for the MACE after ACS [21, 22]. Present study revealed diabetes was not independent predictor of MACE, because there were a few severe diabetic patients such as HbA1c ≥8.0% (24; 5.6%). A previous report from the JAPAN-ACS study showed that the low HDL-C at the baseline was significantly associated with the incidence of cardiovascular events [23]. In current study, multivariate Cox proportional hazards analysis of MACE adjusted as age revealed HDL-C ≤40mg/dL was not independent predictors of MACE.

Nevertheless of these confusing effect, our results indicate the importance of rapid reduction of the LDL-C level in patients with AMI for the favorable outcome. Therefore, to achieve larger percent rapid reduction of LDL-C as recommended by the 2013 ACC/AHA guideline, statin therapy of at least moderate-intensity should be initiated as soon as possible in patients with AMI.

### Limitations

Although this was a prospective, multi-center study, some limitations are still present. First, the sample size was relatively small. Second, we had no data regarding administration of omega-3 unsaturated fatty acid, fibrate and dual antiplatelet therapy through the study. Third, although we had data of patients with multi-vessel, we had no data of PCI for non-infarct-related artery.

## Conclusions

We conclude that rapid reduction of LDL-C level is strongly associated with favorable outcome in patients with AMI.
